# Supplementary material for: The Reindeer Circadian Clock Is Rhythmic and Temperature-compensated But Shows Evidence of Weak Coupling Between the Secondary and Core Molecular Clock Loops
Source: J Biol Rhythms. 2024 Oct 6;39(6):554–67. doi: 10.1177/07487304241283066 (PMC11613641; doi:10.1177/07487304241283066)
Supplement: sj-zip-1-jbr-10.1177_07487304241283066 – Supplemental material for The Reindeer Circadian Clock Is Rhythmic and Temperature-compensated But Shows Evidence of Weak Coupling Between the Secondary and Core Molecular Clock Loops [file sj-zip-1-jbr-10.1177_07487304241283066.zip › Supplementary Tables and Figure Legends.docx]

|  | Primer | Primer sequence (5’-3’) |
| --- | --- | --- |
| SDM mutation primers:  Inserting BamHI cutting site | forward | CGACGGTATCGGATCCCGAGCTCAAGCTTCG |
|  | reverse | CGACGGTATCGGATCCCGAGCTCAAGCTTCG |
| SDM mutation primers: RORE mutation | RORE1 forward | GGGGAGCGGATTGGTCGGAAAGTGTACGTG TGGTGCGACATTTAGGGAAGG |
|  | RORE1 reverse | CCTTCCCTAAATGTCGCACCACACGTACACTT TCCGACCAATCCGCTCCCC |
|  | RORE2 forward | GACATTTAGGGAAGGCAGAAAGTGTACGTGG GACGGAGGTGCCTGTTTACCC |
|  | RORE2 reverse | GGGTAAACAGGCACCTCCGTCCCACGTACAC TTTCTGCCTT CCCTAAATGTC |
| qPCR primers | Mouse Ppib forward | AAGTCACAGTCAAGGTATAC |
|  | Mouse Ppib reverse | TAGCCAAATCCTTTCTCTC |
|  | Mouse Bmal1 forward | CTGAAACACCTAATTCTCAG |
|  | Mouse Bmal1 reverse | CATTCTGGCTATAATTGAGG |
|  | Mouse Per2 forward | CACAAAGAACTGATAAGGAC |
|  | Mouse Per2 reverse | CTGGTAGTACTCCTCATTAG |
|  | Reindeer Ppib forward | CTGAGAATTGGAGATGAAGA |
|  | Reindeer Ppib reverse | GGAATTTGCTGTCTTTGTAG |
|  | Reindeer Bmal1 forward | TGAGTATTTCCATCAAGACG |
|  | Reindeer Bmal1 reverse | ATGAAACTGAACCACCGA |
|  | Reindeer Per2 forward | GCCATCATTATCTGCAAG |
|  | Reindeer Per2 reverse | TCCAGAGGTATTTCTTAGTC |

**Table S1.** Sequences for all primers used. Underlined sequences refer to mutation sequences. Mutation was done with the Site-directed mutagenesis (SDM) kit.

**Figure S1.** Example raw data PMT data (red dots) from an empty chamber showing low amplitude baseline changes under temperature cycles (black dots).

**Figure S2.** All replicates of mouse and reindeer fibroblast transduced with *Bmal1:luc* under different constant ambient temperatures (n=6 cultures for each species).

**Figure S3.** (a) All replicates of mouse and reindeer fibroblast transduced with either *Bmal1:luc* or *Per2:luc* and measured under an ambient temperature cycle. Furthermore, cultures for each respective gene and species were DEX-synchronized 12h apart from each other, effectively resulting in eight experimental groups: Mouse Bmal1 + 0, Mouse Bmal1 + 12, Mouse Per2 + 0, Mouse Per2 + 12, Reindeer Bmal1 + 0, Reindeer Bmal1, Reindeer Per2 + 0 and Reindeer Per2 + 12 (n = 3 for each group). Zoomed-in versions of the baseline-corrected bioluminescence data are provided for *Bmal1:luc* and *Per2:luc* in reindeer fibroblasts after the temperature cycle. (b) Data of peak expression (centre of gravity) for each gene, species and day of the temperature cycle experiments. Data is plotted in circular plots to visualize the phase relationships. Data corresponds to **Figures 2c and 2f**.

**Figure S4.** Three different cell lines (U2OS, mouse fibroblast and reindeer fibroblasts) were either transduced with the *Bmal1:luc* or the RORE-mutated *Bmal1:luc* reporter. (a) Figures in the first row show the raw data plotted on a common axis to visualize absolute *luciferase* expression levels between the reporters. (b) Figures in the second row show the same data plotted on different axes to visualize rhythmicity or the lack thereof. (c) Figures in the third row show baseline corrected data. (d) Figures in the fourth row show all replicates of wildtype and the mutated promoter reporter under the temperature cycle (n = 4 cultures for each respective reporter and cell line).

**Figure S5.** Sequence alignment between the mural *Bmal1* promoter of the *Bmal1:luc* reporter (pLV6-Bmal1-luc) and the corresponding reindeer sequence.

**Figure S6.** Amino acid sequence alignment of RORs and REV-ERBs between mouse and reindeer.
