# Supplementary figures and images for: The Reindeer Circadian Clock Is Rhythmic and Temperature-compensated But Shows Evidence of Weak Coupling Between the Secondary and Core Molecular Clock Loops
Source: J Biol Rhythms. 2024 Oct 6;39(6):554–67. doi: 10.1177/07487304241283066 (PMC11613641; doi:10.1177/07487304241283066)

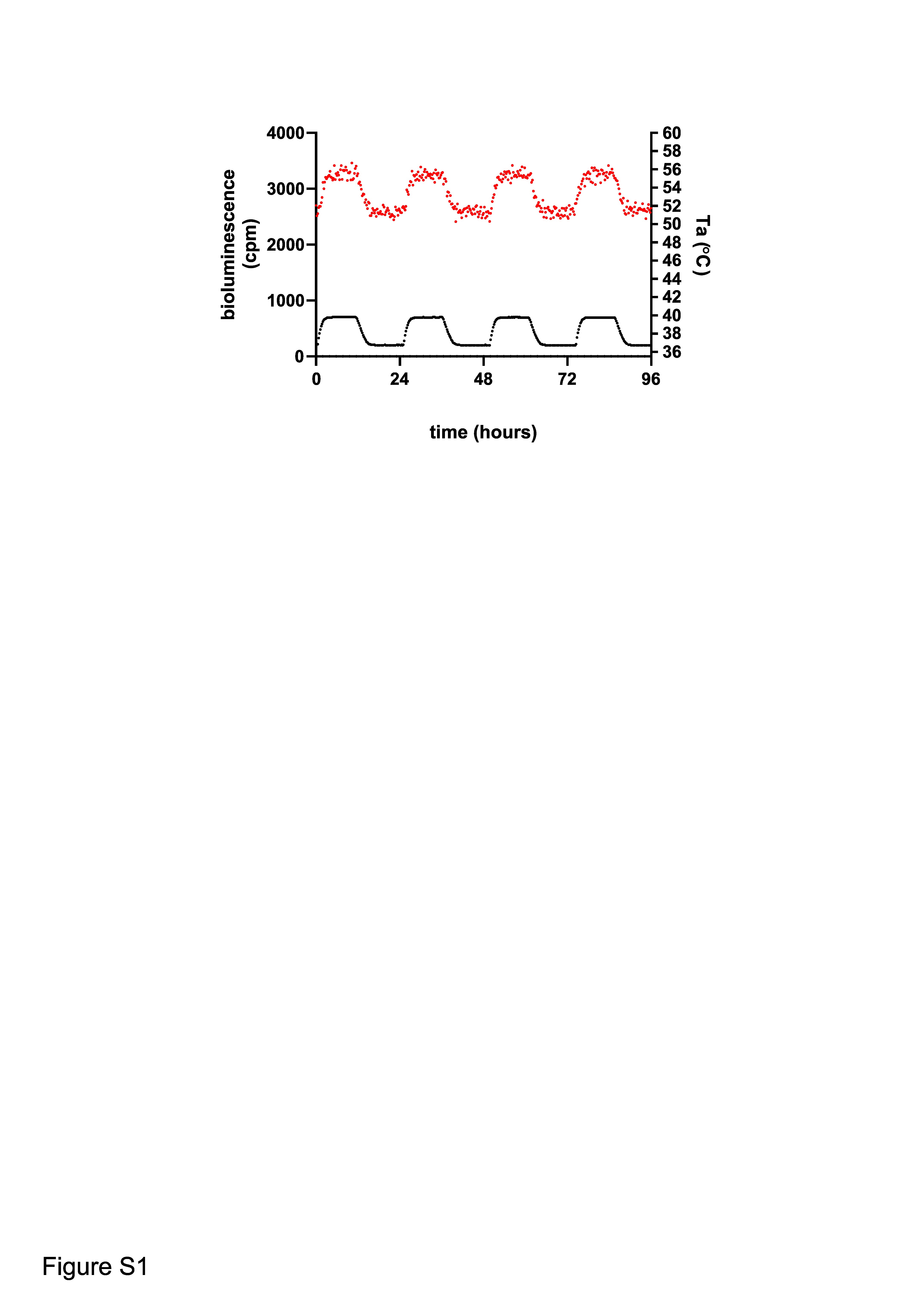

Supplement: sj-zip-1-jbr-10.1177_07487304241283066 – Supplemental material for The Reindeer Circadian Clock Is Rhythmic and Temperature-compensated But Shows Evidence of Weak Coupling Between the Secondary and Core Molecular Clock Loops [file sj-zip-1-jbr-10.1177_07487304241283066.zip › SF1.jpg]

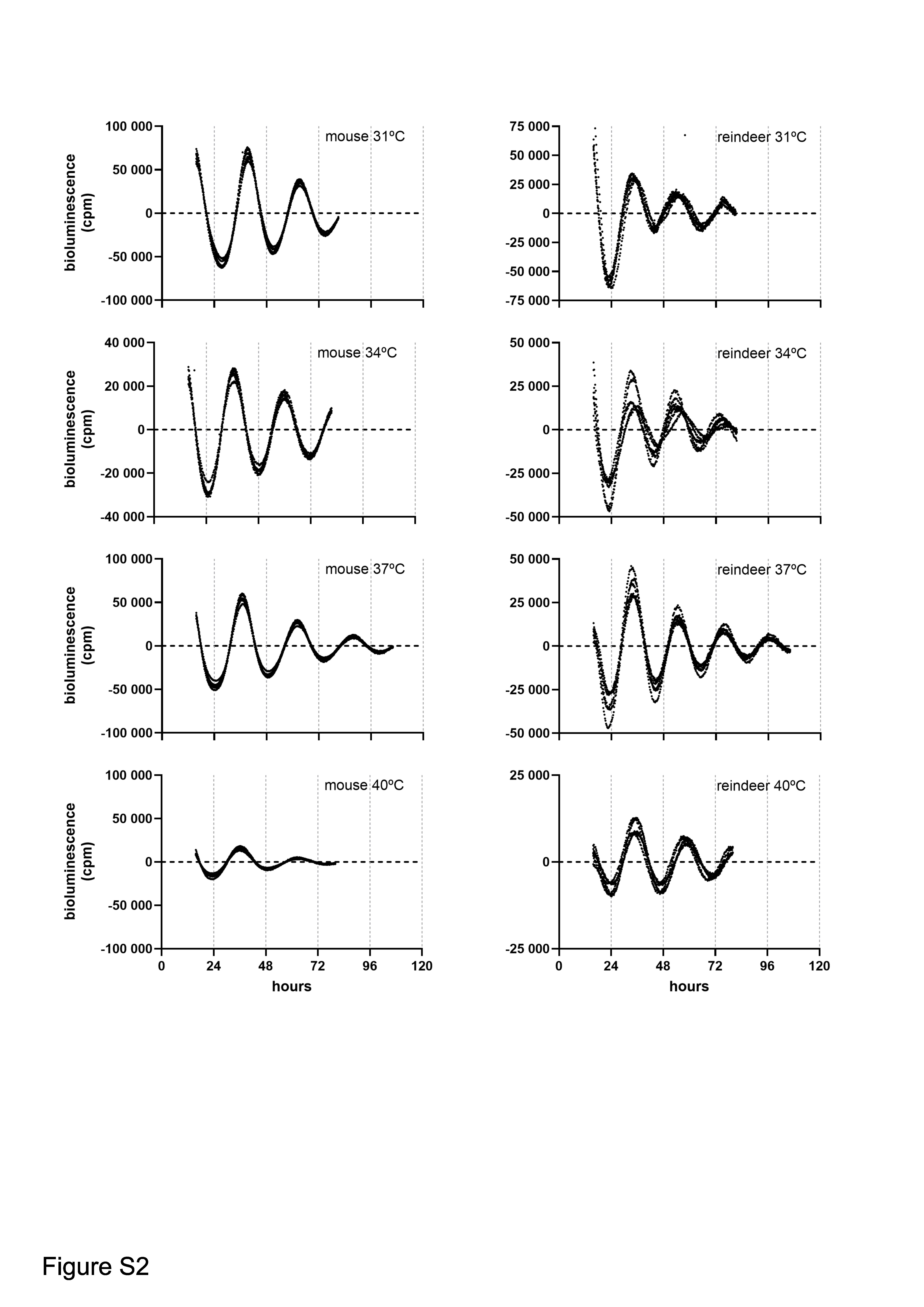

Supplement: sj-zip-1-jbr-10.1177_07487304241283066 – Supplemental material for The Reindeer Circadian Clock Is Rhythmic and Temperature-compensated But Shows Evidence of Weak Coupling Between the Secondary and Core Molecular Clock Loops [file sj-zip-1-jbr-10.1177_07487304241283066.zip › SF2.jpg]

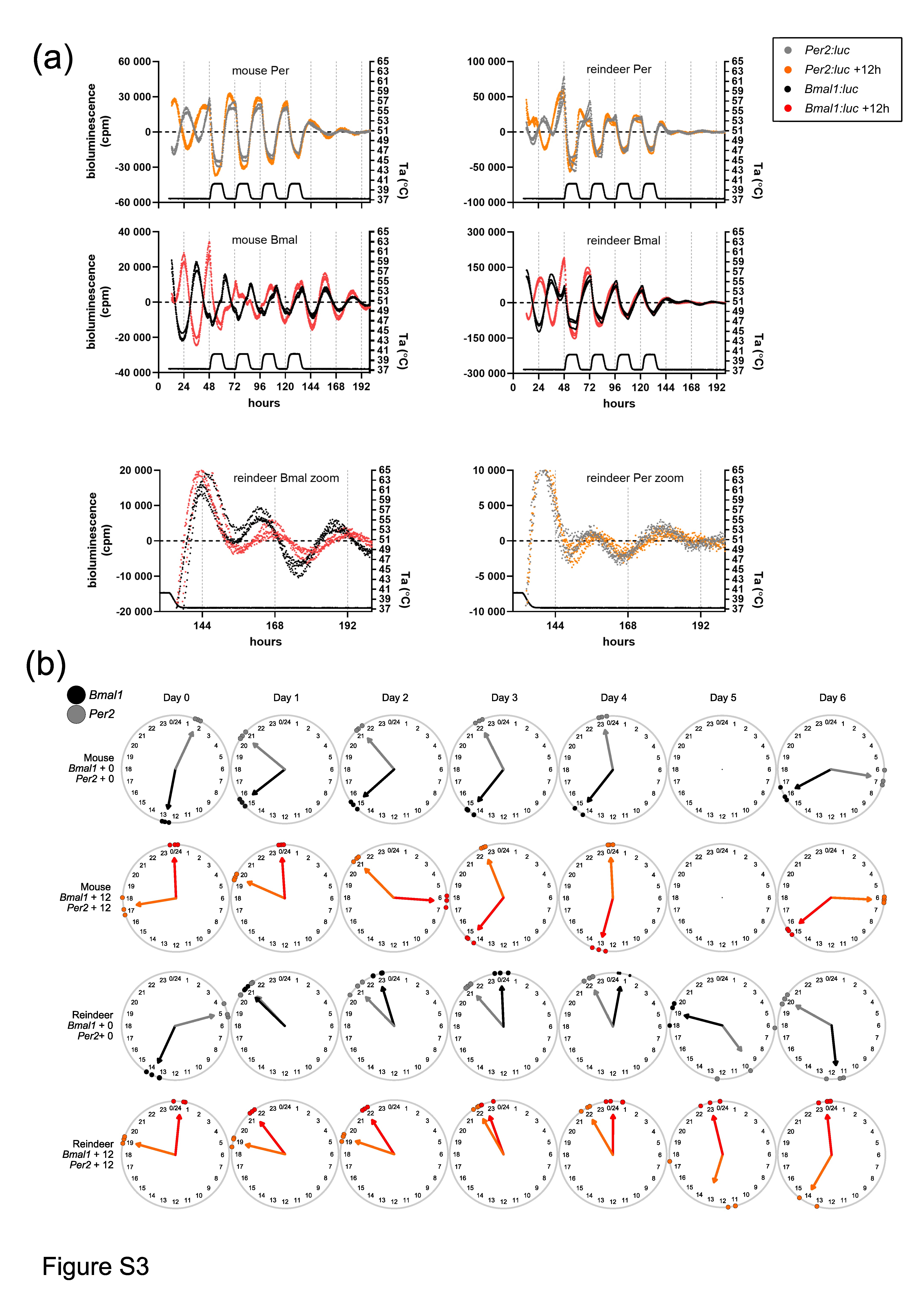

Supplement: sj-zip-1-jbr-10.1177_07487304241283066 – Supplemental material for The Reindeer Circadian Clock Is Rhythmic and Temperature-compensated But Shows Evidence of Weak Coupling Between the Secondary and Core Molecular Clock Loops [file sj-zip-1-jbr-10.1177_07487304241283066.zip › SF3.jpg]

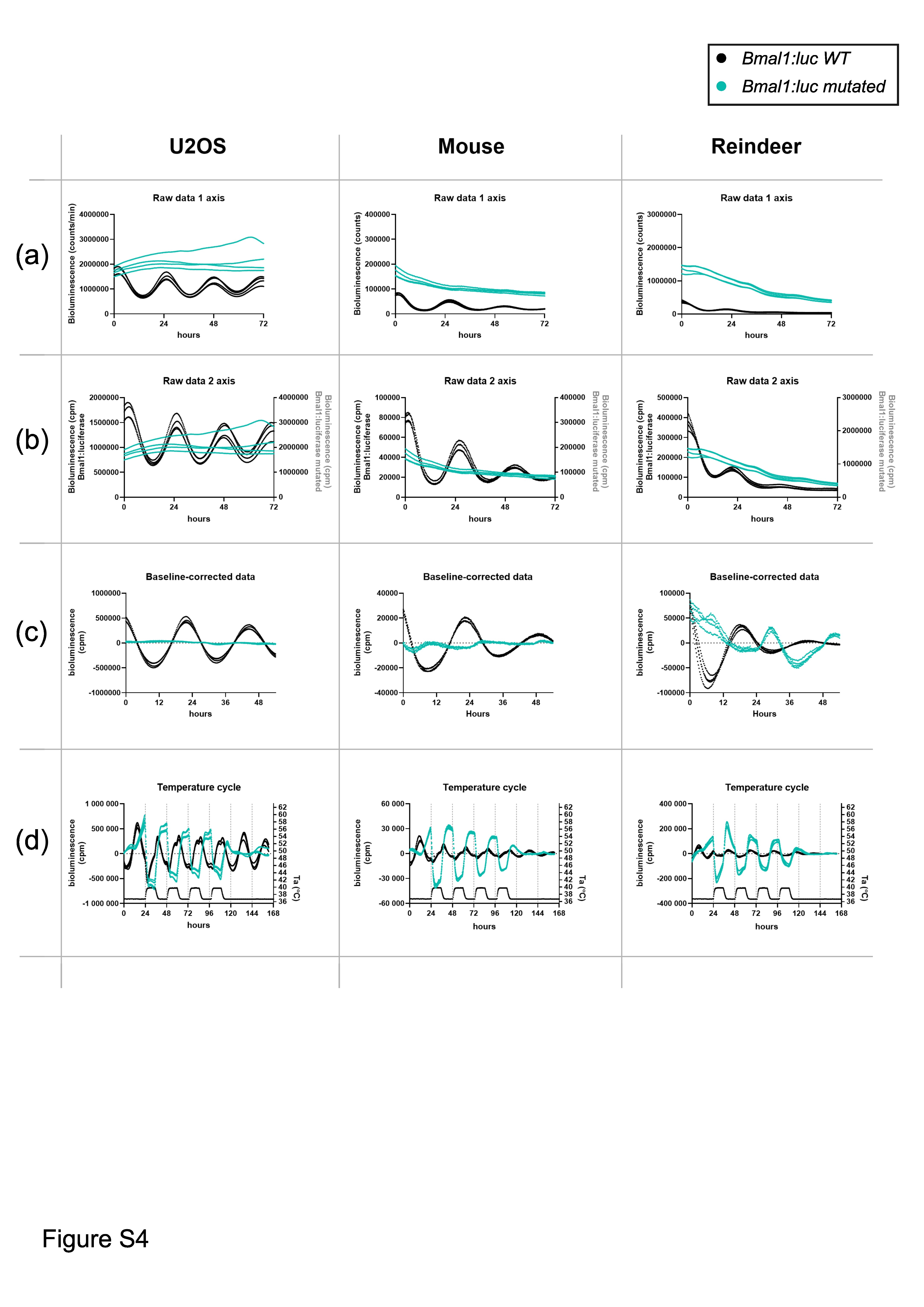

Supplement: sj-zip-1-jbr-10.1177_07487304241283066 – Supplemental material for The Reindeer Circadian Clock Is Rhythmic and Temperature-compensated But Shows Evidence of Weak Coupling Between the Secondary and Core Molecular Clock Loops [file sj-zip-1-jbr-10.1177_07487304241283066.zip › SF4.jpg]

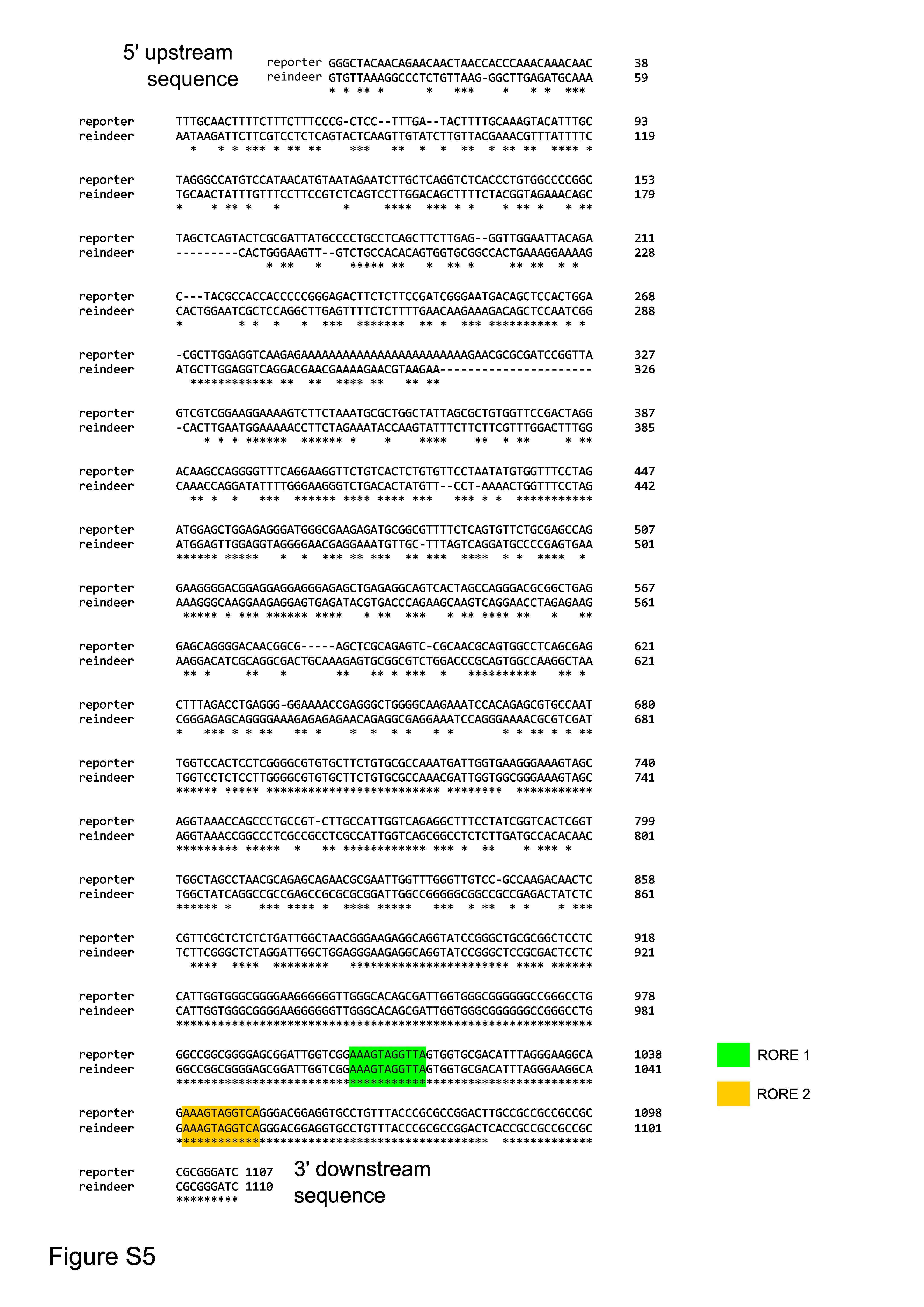

Supplement: sj-zip-1-jbr-10.1177_07487304241283066 – Supplemental material for The Reindeer Circadian Clock Is Rhythmic and Temperature-compensated But Shows Evidence of Weak Coupling Between the Secondary and Core Molecular Clock Loops [file sj-zip-1-jbr-10.1177_07487304241283066.zip › SF5.jpg]

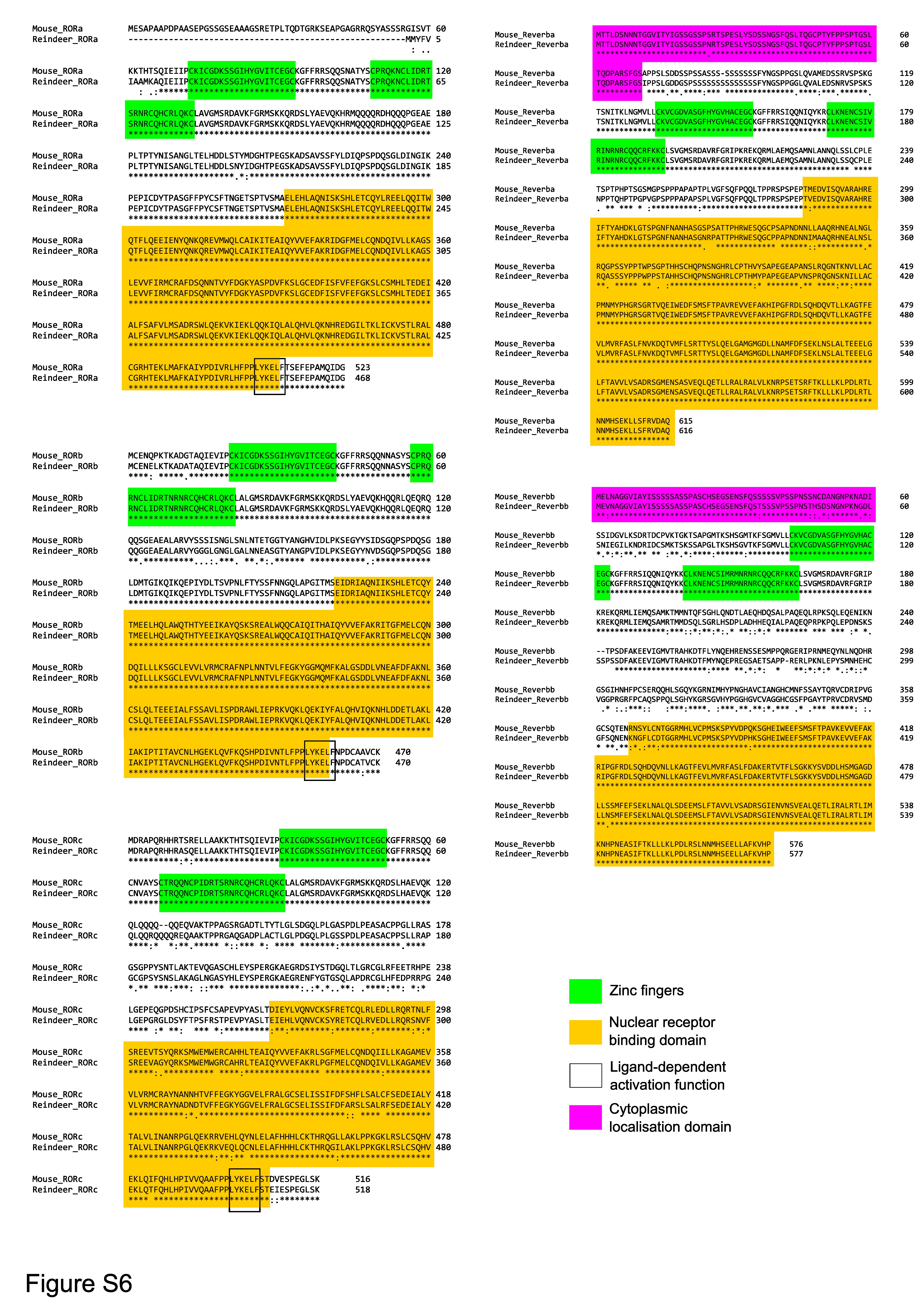

Supplement: sj-zip-1-jbr-10.1177_07487304241283066 – Supplemental material for The Reindeer Circadian Clock Is Rhythmic and Temperature-compensated But Shows Evidence of Weak Coupling Between the Secondary and Core Molecular Clock Loops [file sj-zip-1-jbr-10.1177_07487304241283066.zip › SF6.jpg]
